# Brewing with Starchy Adjuncts: Its Influence on the Sensory and Nutritional Properties of Beer

**DOI:** 10.3390/foods10081726

**Published:** 2021-07-26

**Authors:** Raquel Cadenas, Isabel Caballero, Dieudonné Nimubona, Carlos A. Blanco

**Affiliations:** Dpto. Ingeniería Agrícola y Forestal (Área de Tecnología de los Alimentos), E.T.S. Ingenierías Agrarias, Universidad de Valladolid, 34004 Palencia, Spain; raquel.cadenas@alumnos.uva.es (R.C.); isabel.caballero@uva.es (I.C.); nimurukundo@gmail.com (D.N.)

**Keywords:** brewery, beer, beer adjuncts, starchy beer adjuncts

## Abstract

In brewing, the use of cereals (wheat, barley, maize, rice, sorghum, oats, rye or millet), pseudo-cereals (buckwheat, quinoa or amaranth) and tubers (sweet potato), as starch adjuncts, is being promoted for the production of a variety of high-quality beers, from sensory and nutritional points of view. The sensory properties of the obtained beer depend on the characteristics of each adjunct but also on the forms in which the adjunct is added: whole cereal, grits, malted, extruded grains, torrefied and syrup. Among these common forms, the extruded grains (maize or rice) produce a higher content of aroma compounds in beer. From a nutritional point of view, the use of non-conventional starch adjuncts, such as black rice, buckwheat or sweet potato, leads to an increase in the polyphenol content of the beer, and thus, its antioxidant capacity. Cereals such as maize, rice, sorghum or millet are the most promising for the production of gluten-free beers. A close relationship can be developed between the use of adjuncts in the beer industry and the use of commercial enzymes. Advances made by biotechnology to design new enzymes with different functionalities could be associated to a future increase in adjunct usage in brewing.

## 1. Introduction

Cereals are widely used to produce alcoholic beverages such as beer, vodka, whisky, bourbon and others. Alcoholic beverages produced from maceration and fermentation of cereals are extensively used and appreciated worldwide for their taste and flavour [1].

Beer is a classic alcoholic beverage and is one of the most popular internationally [2]. The raw materials and the several procedures that take place during brewing (milling, mashing and fermentation) are responsible for beer being nutritionally rich in amino acids, carbohydrates, vitamins, minerals and phenolic substances [3]. The principal polyphenols that can be found in beer include phenolic acids, flavonoids, tannins, proanthocyanidins and amino phenolic compounds, and originate principally from malt and hops, contributing considerably to the colour, flavour and stability of beer [4].

Multiple epidemiological research studies report that a moderate consumption of beer decreases cardiovascular mortality due to changes in the lipid profile and boosts antioxidant capacity. Furthermore, both regular and moderate beer drinking is associated with a lower risk of Alzheimer’s disease, diabetes and osteoporosis [5,6,7,8]. In moderate beer consumers, a 20–25% decrease in the mortality rate from coronary heart disease has been found [9]. All these benefits are attributed to the nutrients from the raw materials (water, malt, hops and yeast) and those created during the brewing process itself.

Beer is generally produced from barley malt, although other malted or unmalted cereals are also used in association with or instead of barley malt [4]. These ingredients are called adjuncts and their use can improve the levels of bioactive compounds and give new organoleptic characteristics to beer.

Adjuncts are substances, apart from malt, which are used as a source of extract. They are used both because they generate a much cheaper extract than malt and/or because they provide beneficial characteristics to the product. For example, they can attenuate the levels of soluble nitrogen and polyphenolic tannins in the wort, favouring the use of high nitrogen malts (rich in protein) and the brewing of a beer less susceptible to turbidity. The higher the percentage of adjuncts in the mash, the more difficult it is to achieve good extract recoveries and, in addition, it tends to increase wort viscosity, decrease drainage and decrease fermentability. The addition of soluble sugars to the wort increases the capacity of the brewhouse and is an easy method to produce high gravity wort and regulate wort fermentability [10].

In addition to these advantages, the use of adjuncts for partial replacement of barley malt makes it possible to take advantage of availability of raw materials on the local market and often reduces production costs [11,12]. For example, the use of 30% maize as an adjunct has been shown to lead to an 8% decrease in total beer production costs [11].

Commercially available oat (*Avena sativa* L.) and sorghum (*Sorghum bicolor* (L.) Moench) flours are employed in the brewing process to decrease mashing times due to the high solubility (extractability) of the finely grinded cereals, and thus, reducing energy requirement and costs [13].

For all these factors, the use of adjuncts in brewing is a growing market in the United States of America and Europe [14] and they are currently included in the brewing of 85–90% of beers produced globally [15].

Adjuncts are generally considered to be alternative sources to barley malt that contribute fermentable sugars to the brewing wort. This definition includes non-malted solid raw materials, liquid adjuncts and malted cereals other than barley [15,16]. Trace elements, such as iron, copper and zinc, are relevant due to their role as cofactors in metabolic and biosynthetic processes, such as beer fermentation [17].

## 2. Adjunct Classification

**Figure 1:**
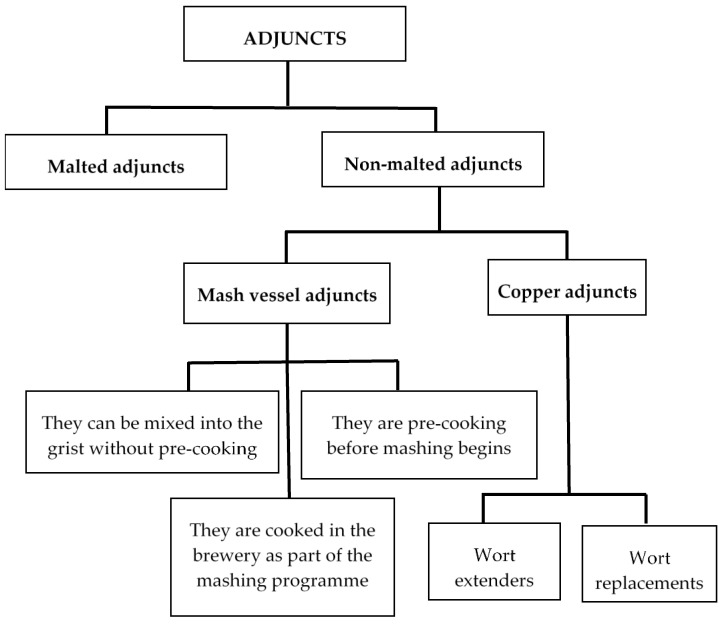
Classification of adjuncts used in brewery.

There is a huge variability of adjuncts, and they can be classified according to different criteria (Figure 1). Depending on the state they are in, they are classified as in the following sections.

### 2.1. Solid Adjuncts

These include unmalted cereals, unmalted pseudocereals and derivatives and granulated sugar [18]. In addition, according to the generic composition, malted cereals other than barley are considered within this group. In general, solid adjuncts may have a variety of presentations: whole grain, semolina, flour, flakes, roasted or malted (in the sense of malt that is distinct from the normal malt employed to brew the style of beer in question) [19].

### 2.2. Liquid Adjuncts

They are commonly aggregated at the boiling phase of the wort. They commonly contain sugar cane or sugar beet derivatives, sucrose-based syrups and starch hydrolysed syrups, including malt extracts and syrups from hydrolysed cereals [18].

They may also be categorised in accordance with the time at which they are incorporated in the beer production process [20]. Malted adjuncts follow the malting process, which is prior to the brewing process, while unmalted adjuncts can be added at different stages of the brewing process.

Accordingly, non-malted adjuncts can also be classified as follows:

Mash vessel adjuncts: adjuncts that undergo hydrolysis in the process of mashing with the malt or by external microbial enzymes [18]. This group includes the amylaceous adjuncts, among them:

Products that are not processed, but which may be mixed together with grinding, in the same way as wheat flours [18,21].

Products processed outside the brewery, including cereal flakes, micronised and torrefied whole grains and pearled cereals [18,21].

Unprocessed products requiring cooking in the brewhouse during the mash process, such as semolina and flours of rice, maize or sorghum or derived refined starches of such ingredients [18,21].

This classification is related to the variation in gelatinisation temperatures of starches produced from a variety of origins. In the instance of adjunct starch, gelatinising at hotter temperatures compared to malt enzymes, a previous gelatinisation of the starch is required prior to mixing the adjunct with the malt wort mash [11,22]. This may be achieved by using a grain boiler, an auxiliary kettle in the brewhouse, if mashing by infusion is employed. Gelatinising of the raw grains can be carried out in the mash tun before adding the malt grains with water. However, this procedure extends wort production [22].

Copper or kettle adjuncts: do not require hydrolysis and may be directly incorporated into brewing wort (boiling stage) [18,21].

They can be divided into two categories. First, wort extenders, mainly adding only carbohydrates (such as sucrose, invert sugar and hydrolysed starch syrups), and wort replacers, such as malt extracts and hydrolysed cereal syrups. These substances add carbohydrates and a complex variety of other components to the process stream.

Table 1 provides an overview of the main types of adjuncts commonly found in the global beverage industry today [19]. Obviously, the variety of adjuncts normally present in a brewery varies according to its particular localisation.

Due to the wide variety of adjunct types used in the brewing industry, this work focuses on starchy adjuncts. The effects of starch adjuncts to the organoleptic properties and nutritional features of the finished product will be analysed.

## 3. Barley Grain and Other Brewing Cereals: Starch Structure

Barley is the principal grain used in the production of beer [23]. The complexity of genetic and physico-chemical features and their correlated relationships has resulted in ongoing research to increase the quality of barley cereal [24]. The most important characteristic of the grain quality is its size, as maltsters prefer fat kernels [25].

Table 2 shows the variability in starch, amylose, amylopectin and protein content in cereal grains.

Starch is a determinant of grain quality in cereals; however, the relationship between variations in starch structure and its impact during processing have not yet been fully explored. Recently, Balet et al. have examined the starch properties of South African grown malting barley varieties [27]. In this study, no differences in amylose content were found; however, variation in the amylose chain length distribution was observed, although all barleys showed similar granule parameters. The longer amylose chain length resulted in increased pasting temperature. The variation in starch structure was not observed when measuring content and could impact fermentation efficiency through a variation in fermentable sugars hydrolysed from the starch [27].

The starch content of barley is lower than that of other cereals (Table 1). Independently of the starch level, usually between 55% and 60%, several starch variables, such as amylopectin and amylose content or the amylopectin:starch amylose ratio, are the result of starch structure variation, such as the rate of branching of amylopectin, and the length of the amylose chain [28].

Regarding the selected barley for malt quality, large kernel size and protein content (between 9% and 13% on a dry basis) are particularly relevant variables. Starch content was significantly associated with kernel size, reflecting that larger barley kernels usually contain significantly more starch and larger molecular sizes of amylose and longer amylose chains [29].

Fermentable sugars may be complemented with solid or liquid adjuncts. These generally require some processing, i.e., particle size reduction and starch gelatinisation, before their addition to the mash [10]. Some adjuncts may provide more further enzymatic activity. However, the hydrolysis of the adjunct starch required for fermentation is almost entirely dependent on the enzymes in the malt.

There is significant variation in starch, amylose and amylopectin content, depending on the cereals used in beer production, and there is also variation in the starch structure in these cereals of similar proportions for amylose chain length, degree of amylopectin branching and amylopectin chain length. It is interesting to highlight that amylopectin is the predominant constituent in cereals, with three to four times the amount of protein [30].

## 4. Brewing with Adjuncts

Brewing with unmalted cereal adjuncts is a major challenge for the beer industry; therefore, a more detailed understanding of the factors which limit the upward incorporation rates of unmalted adjunct materials is necessary.

When high adjunct levels are added, the brew functionality and processability of the mash must be achieved, and there must be no adverse impacts on the quality of the beer product [31]. The principal liability in terms of processability when non-malted adjuncts are added is the lowering of amylolytic, cytolytic and proteolytic enzyme activities in the mash, as these enzyme systems are excited and synthesised during the mashing operation. The action of these three enzyme systems occurring during malting and mashing impacts on the chemical constitution of the wort and the effectiveness of the recovery of the brewing extract [32]. The deficiency in enzyme activity and variations in the composition of unmalted adjuncts can impact the flavour profile of beers with adjuncts [18,33]. However, these impacts on the aroma and flavour of finished beer have not yet been studied in depth [33].

With the above in mind, the use of adjuncts in beer production should be considered. Its use normally impacts the first stages of the production process.

After malting barley has been milled, other cereals that can be used in the process can also be added at this time, if they are not already milled. Adjuncts such as maize, rice, wheat, unmalted barley or sorghum are all suitable choices available to beer production. Nevertheless, if the adjunct starch has a gelatinisation temperature (Table 3) exceeding the optimum β-amylase activity temperature of about 62 °C, as is the case with maize, rice and sorghum, it has to be gelatinised first and boiled separately from the wort in a cereal cooker [34]. If the adjunct is in a non-grinding form, such as pregelatinised flakes or syrup, it is added at a further step in the process.

Afterwards, biochemical changes, such as starch gelatinisation and starch-degrading enzyme activity, occur during mashing [30]. The two critical objectives of these steps are to maximise gelatinisation and the subsequent hydrolysis of starch into fermentable sugars. Both processes are complex and rely on a great number of mashing variables [36].

The enzymes provided by the barley malt are very active on starch and proteins during mashing. Unmalted grains will impact the mashing times and temperatures used during the mashing process.

Different parameters influence the rate of starch hydrolysis during mashing, such as temperature, water quality, grist size and pH and duration of mashing. The gelatinised starch is required for enzymatic hydrolysis and thereby has an important role in starch conversion [37]. Several mash time and temperature ranges exist; however, they all have the main goal of gelatinising the starch and optimising the enzymatic activity [38].

Temperature is the most significant variable in the mashing process. Understanding its importance is critical in predicting the performance of a specific mash [30]. Raising the temperature of the mash can be advantageous, as it speeds up the rate of all enzymatic reactions, decreases viscosity, gelatinises the starch and speeds up diffusion and dissolution [10]. However, this rise in temperature will also speed up the denaturation of all enzymes, and the rise in dissolution rate may lead to the extraction of undesirable substances (e.g., tannins) in the malt. Hence, the temperature of a mash must be high enough to achieve fully gelatinisation, but also low enough to not degrade the different amylolytic enzymes very quickly [36].

The pH of the mash (generally meaning the initial pH of the mash, as pH is rarely, if ever, controlled during the mash process) is also fundamental to the performance of the mash, but its impact is less well known. This gap in knowledge is magnified by the fact that acid dissociation rises with temperature, thereby the measured pH value of a sample is dependent on the temperature of the sample during measurement [10]. In general, the pH of wort is in the 4.6–5.8 range [36].

A pH of around 5.5 is kept during the mashing process to ensure optimised amylase activity. The pH control while mashing will play a critical role in mashing time and grain conversion, and monitoring should be done when non-malted grains are used.

The use of adjuncts does not greatly impact the other steps in the beer production process.

Relevant features in the wort are nitrogen content (total soluble nitrogen, TSN) and composition (free amino nitrogen, FAN), pH and the concentration and composition of fermentable sugars. All these attributes are developed in the mash and have an influence on fermentation. The use of unmalted grains impacts all these attributes [39].

## 5. Starch Adjuncts Used in Brewing and Their Effects on the Sensory and Nutritional Properties of Beer

The Food and Agriculture Organization (FAO) reports that there are 11 starch adjuncts among the cereals and pseudocereals that have a wide application in brewing. Cereals include wheat, barley, maize, rice, sorghum, oats, rye and millet and pseudocereals include buckwheat, quinoa and amaranth [22].

In particular, sweet potato (*Ipomoea batatas*) is included in this review, since it is composed mainly of starch and sugars in 80–90% of its dry matter and has excellent properties for use as an adjunct in the brewing industry [14,40].

A summary of starch adjuncts used in the brewing industry, their correlation with beer type and the general effects on the sensory and nutritional properties of beer are presented in Table 4.

### 5.1. Relevant Attributes for the Use of Adjuncts

Switching between different types of adjuncts is a challenge. The risk of altering the nature of the beer aside, adjunct changing from one to another may involve modifications to the brewery’s equipment. For example, the required brewhouse handling plant for syrups is totally distinct from that needed for any mash tun adjunct, and the equipment required to handle flours, flakes and grits are all distinct.

Each adjunct has a number of characteristic attributes. It is essential to analyse the attributes of each adjunct for use in brewing and to see if they are comparable to those of barley malt, which is the main grain used for brewing beer.

As they are starchy adjuncts, they will provide starch as a source of fermentable sugars. Starch is composed of two polymers, amylose (unbranched α-D-glucose chains) and amylopectin (branched α-D-glucose chains). Starch hydrolysis leads to the the production of fermentable sugars (glucose, maltose and maltotriose). Further starch hydrolysis efficiently involves the activities of α-1,4-branching and α-1,6-branching hydrolases, with endo-amylases (α-amylases) and exoamylases (β-amylases) breaking only α-1,4 bonds, and glucoamylases and α-glucosidases exhibiting α-1,4-branching and α-1,6-branching activities [59,60].

It is essential to know the grain’s extract percentage and its gelatinisation temperature range [16]. The higher the percentage of the extract, the higher the yield of grain to be used. The range of gelatinisation temperatures will be important for the mashing stage, since depending on the range, the grain can be mashed together with the malt (as is the case for wheat or barley) or needs to be pre-processed in another vessel (rice or corn) [16].

The addition of adjuncts in the mash kettle normally leads to a lowering of the extract content within the wort. Reduced levels of malt in the wort produce a lower set of enzymes engaged in the hydrolysis of malt constituents, such as starch or proteins and cell wall constituents [61,62]. Nevertheless, in high-quality malt, there is a small surplus of enzymes, which may also degrade the compounds introduced by the inclusion of adjuncts [18]. In case of higher amounts of malt replacement, the usage of external enzyme compounds is required.

Starch gelatinisation performed at higher temperatures compared to traditional mash also facilitates starch decomposition by amylolytic enzymes. The utilisation of wheat, oats or raw barley in quantities above 20% with no application of external enzymes leads to a lower extract content of the wort and reduces the final alcohol content of the beer as a result [13,62]. Enzyme formulations can offset this problem.

Most grains have gelatinised starch in the range of normal high-temperature mashing (63–70 °C) (Table 3). When starch granules puff up and break down, they become susceptible to rapid enzymatic attack (i.e., gelatinises) at temperatures low enough for the malt enzymes to remain active, and therefore, they will not require precooking (e.g., wheat flour). However, if the starch has a high gelatinisation temperature (e.g., corn), the material needs to be precooked at a high temperature so as to gelatinise the starch (either by shelling or in a lauter in the brewery) before mixing it with the principal malt wort at a temperature at which the malt enzymes can be active.

In terms of relevant malt attributes, the importance of malt extract yield should be emphasised. Amylolytic enzymes are formed during malting, which break down starch to simple and soluble sugars. Fermentable extract generated during mashing plays an essential role in the success of fermentation. In the case of barley malt, the percentage of extract is around 80% [63].

The β-amylase is most commonly of vegetal origin; however, some microbial β-amylases have also been described. The β-amylase can be synthesised by bacterial strains belonging to the *Bacillus*, *Pseudomonas* and *Clostridium* spp. [64] and by fungal strains belonging to *Rhizopus* [65] and by *Volvariella volvacea* [66]. Some crops such as soybean, sweet potato and barley have a high content of β-amylase [67]. One of the most sought-after characteristics of amylases is thermostability, which has a role to play since starch starts to solubilise at high temperature (100 °C) and in acidic conditions (pH 4.5–5.5). Various microbes have been previously documented to produce thermostable enzymes, especially amylases, which can be active at high and low pH as well.

The β-amylases split two linked glucose molecules on the reducing end of the chain and the α-amylases randomly hydrolyse the α-1,4 bonds of starch. The α-amylases are the highest thermostable starch-degrading enzymes [63,67].

A final significant attribute to consider is the free amino nitrogen (FAN) content. FAN values must be high enough to guarantee that a lack of nitrogenous nutrients for the yeast does not limit fermentation. FAN should also not be too high, as a large concentration can promote the development of off-flavours due to Maillard reactions [63].

The ingredients used as adjuncts and their sensory and nutritive effects on beer are analysed below.

### 5.2. Cereals

#### 5.2.1. Wheat (*Triticum aestivum* L.)

Wheat is one of the cereals with the longest history of being used as a raw ingredient for the elaboration of malt and beer. It is used in the brewing of many styles of beer [63]. As wheat grains are unhulled, they soak up water more quickly and have a higher diastatic power than barley, which allows for shorter mashing times. In addition, wheat malt has a higher α-amylase activity, a higher extract content than barley and is usually rich in FAN, which helps the proper development of fermentation [16]. As for the use of unmalted wheat, it has been found that at low proportions (less than 20%), despite slightly reducing the FAN content, better quality beers are obtained [42]. For these reasons, malted and unmalted wheat grains (wheat flour, torrefied wheat or wheat starch) are widely used in the brewing industry. In line with grains that have similarities to common wheat is the Triticale grain. Triticale grain is a hybridisation of wheat (*Triticum aestivum* L.) with rye (*Secale cereale* L.) and its malt is characterised by high diastatic power, short saccharification time and high extractability [68]. These properties mean that Triticale malt shows high potential to be used as a substitute for barley malt for the production of European Lager beers [69].

In general, wheat beers, usually ale beers, have a strong malt component and generally exhibit a much greater aromatic flavour than lager beers. One of its components, 4-vinylguaiacol, is responsible for phenolic aromas [63]. As for wheat malts (Pale, Roasted and Smoked), they produce generally slightly darker beers [16], with greater intensity of flavours and odours, and with more body than beers brewed with barley malt [41]. Other distinct changes are perceived with the use of unmalted wheat. For example, the use of wheat flour results in clearer beers, higher alcohol content and lower foam stability compared to beers brewed with 100% barley malt. In addition, sensory parameters are modified, with a stronger grain odour, more body, less astringency and less bitterness than in 100% barley malt beers [42].

From a nutritional point of view, malt from old wheat varieties, such as Einkorn, has been shown to have higher polyphenol content and greater antioxidant activity than newer wheat varieties, such as common wheat, compared to unmalted barley and moderately lower than barley malt, making it a raw material for producing beers with higher antioxidant activity [70,71].

#### 5.2.2. Barley (*H. vulgare* L.)

The main drawback of unmalted barley is that the unmalted grain is rough and hard to grind and results in a significant proportion of thin material, which leads to significant challenges at filtration [16]. To minimise this drawback, pretreatments are often applied to the grain to gelatinise the starch (e.g., dehulling or extrusion) that facilitate the extraction of β-glucans and pentosans during mashing [22,72] and improve filtration after mashing [73]. On the other hand, a high level of barley malt substitution with unmalted barley usually leads to an inadequate number of enzymes in the mashing process, which are necessary for the hydrolysis of starch, proteins and β-glucans. Therefore, enzyme blends are used to balance the decreased enzyme level [16] and it is feasible to use unmalted barley in brewing if enzyme blends are used.

From a nutritional point of view, the use of 100% unmalted barley in brewing results in a clearer end product, with differences of more than 2 EBC (European Brewery Convention) units, being lighter, with less body and mouthfeel and better foam stability, similar to barley malt [32,43]. Although the organoleptic characteristics are very similar to those of barley malt, when using unmalted barley at proportions of 90%, detectable changes in bitter taste (more astringent and abrasive) are observed.

Lower proportions of unmalted barley (between 50–75%) improve the oxidative stability of the beer. In particular, a lower development of ageing compounds (3-methylbutanal, 2-methylbutanal) is observed after one month of storage [44].

#### 5.2.3. Maize (*Zea mays* L.)

Maize is widely employed as an adjunct in the brewing process to boost the quality of wort and beer. Maize is a great source of useful yeast carbohydrates. In addition, its price decreases in the overall production process with its addition because it is an inexpensive raw material [42]. Regarding its attributes for use in brewing, maize has a high starch gelatinisation temperature and slightly low α-amylase activity (several times higher than that of sorghum, but lower than that of rice malt) [74]. Since the malting process of maize is difficult and expensive and, in fact, not very widely used in brewing [35], maize semolina or refined starch is the most commonly employed form when maize is used as an adjunct [16]. Maize can be used as an adjunct in brewing in many ways: as cereal, flour, starch, expanded, groats, extruded, corn syrup, etc. [75].

From a nutritional and sensorial point of view, corn adjuncts generally add specific aromas to popcorn and sweet corn-like beers due to the presence of 6-acetyltetrahydropyridine, 2-acetyl-1-pyrroline and its analogue 2-propionyl-1-pyrroline [74]. According to a study published by Diakabana et al. in 2013 [7], the use of corn malt results in beers with lower alcohol content, with slightly bitter taste and lower foam stability than beers brewed with barley malt, due to the high level of unsaturated fatty acids in the maize. Maize also influences the colour and flavour of the beer. In fact, beer colour decreases by one EBC colour unit for each addition of 10% ground maize in brewing [11]. Other types of adjuncts, such as corn flour, in proportions below 20%, have a positive effect on beer quality, and sensory aspects such as grain odour, sweetness, bitterness and aroma are better valued in beers with corn adjuncts than in beers without corn adjuncts [42].

The use of corn as an adjunct reduces slightly the total polyphenol content [9,74]. As reported in 2011 by Fumi et al. [9], beers containing corn as an adjunct provide 10–20% of the total recommended daily intake of phenols (1 g/day) and account for about 4–8% of the total antioxidant capacity of the diet. Beyond traditional maize malts, the use of malts from pigmented maize varieties (e.g., Chalqueño variety) results in beers with higher anthocyanin (cyanidin-3-glucoside or catechin) content than those brewed from non-pigmented maize varieties, especially if caramel blue maize malts are used, probably due to their higher melanoidin content [45].

#### 5.2.4. Rice (*Oryza sativa* L.)

Rice is one of the most important cereals used as an adjunct in brewing. There is currently a wide range of rice varieties, each of which having certain characteristics, and not all varieties are appropriate to use in brewing. For example, short-grain rice is preferred because medium- and long-grain varieties can lead to viscosity problems [46]. In the current brewing industry, rice is mainly employed as an unmalted adjunct in association with barley malt, as it can substantially boost the extract content in the mashing phase [35,46]. The difference in structure and constitution between rice and barley makes it necessary to optimise the conditions for malting and mashing. Indeed, rice malt exhibits a poor diastatic power (DP) and a wide gelatinisation temperature. In addition, the amylolytic activity of rice is significantly lower than that of barley and, because of its elevated content of free unsaturated fatty acids, rice is most sensitive to oxidation, with the potential to develop a stale odour [35]. Due to these factors, it is most suitable to use rice as an unmalted adjunct rather than rice malt, and the most used types of rice adjunct are rice semolina, flaked rice, extruded rice, rice flour and rice starch [46].

Rice provides a balanced aroma and neutral taste, and its unmalted adjunct in brewing yields light, dry beers with pleasant flavours [46]. In addition, beers with a high unmalted rice content have a higher colloidal stability [20,22]. Rice malt beers have an alcohol content similar to that of barley malt beers (3.5–5.1%). Sensory tests reveal a pale yellow colour and a thick head with a slight vanilla flavour. The rice malt beer’s sensory profile is comparable to that of malted barley beer in terms of aroma, flavour and mouthfeel, although flatter [46]. This flat sensory profile can cause rice beers to be of little interest from a sensory point of view. A study conducted to try to improve this profile was published by Ceccaroni et al. in 2018 [47]. Brewing beers from special rice malts, such as Caramel and Dark malts, results in beers similar in colour to beers brewed with caramel and chocolate barley malts (Caramel Malt and Chocolate Malt) and also enhances the presence of malt, caramel and vanilla aromas, resulting in a less flat sensory profile.

Regarding the nutritional and sensorial properties, traditional beers from rice malt commonly consumed in India have been shown to exhibit strong antioxidant activity due to their high content of six phenolic compounds (gallic acid, catechin, caffeic acid, p-coumaric acid and salicylic acid). These compounds have anti-tumour, anti-diabetic, anti-allergic, anti-cancer and anti-inflammatory effects [76], and according to the study by Handique et al. [77], the consumption of this type of beer in moderate amounts can contribute to the well-being of humans. In addition to rice malt, the use of rice as an adjunct also contributes nutritional benefits to the final beer. A study by Zhang et al. published in 2019 showed that the use of extruded black rice provides beer with higher essential amino acid contents than those present in Lager (pale or dark) and Ale beers, especially in valine and threonine.

#### 5.2.5. Sorghum (*Sorghum bicolor* L.)

In Africa and Asia, sorghum is used to produce traditional alcoholic and non-alcoholic beverages; alcoholic beverages such as impeke, kaffir beer, tala, burukutu, sorghum wine and pito and non-alcoholic beverages such as kunuzaki are popular examples [78]. The importance of sorghum as a brewing adjunct was recognised during World War II [78]. The use of sorghum as an alternative to barley malt is important in the production of all types of beers (Ale and Lager) [16,48,49,79]. Some problems exist in brewing with sorghum malt, related to its poor diastatic activity (not enough for fully saccharification), elevated gelatinisation temperature and reduced FAN content. Sorghum exhibits low β-amylase activity, but significantly higher α-amylase activity than barley malt. This results in a low production of fermentable sugars and a high dextrin content, which leads to an increase in viscosity. For these reasons, the production of beers brewed with high-quality sorghum malt requires the addition of exogenous enzymes and is not economically viable [13,80]. Typical sorghum grain processing results in mixtures of pearled sorghum, sorghum flour and sorghum meal that are used together in processing [16]. Sorghum meal or extruded sorghum can also be used as a supplement only [81,82].

In relation to nutritional and sensory properties, sorghum malt beers, traditionally produced in Africa, have a relatively low alcohol concentration, a slightly sour taste due to lactic acid formation and give a slight bitter or astringent sensation [22,79,83]. In particular, in the case of sorghum, some sensory differences can be observed in beer depending on whether red or white sorghum varieties are used. Red varieties have a higher tannin content, which contribute more astringency and bitterness, while white varieties contribute sweeter, corn-like flavours [74]. As for unmalted sorghum, the sensory quality of lagers containing up to 50% sorghum grain is similar to that of beers brewed with 100% barley malt, although they have lower foam stabilities [13,81,84]. Schnitzenbaumer et al. published a study in which they brewed two beers with 40% unmalted sorghum (one red and one white variety) and, in both cases, obtained very similar ratings to the 100% barley malt beer in sensory aspects such as aroma, flavour, body and bitterness [84]. Sorghum is a great source of proteins, B group vitamins, minerals and health promoting constituents, such as antioxidant phenolics, fibres and cholesterol-lowering waxes [85].

#### 5.2.6. Oats (*Avena sativa* L.)

Oats are mainly used as an adjunct in Stout brewing [61] and have recently gained importance because they provide beers with unique organoleptic characteristics and can be used to produce beers suitable for coeliacs [49,63]. Oat malt has a lower diastatic power (DP), gelatinisation temperature and α-amylase activity than barley, as well as high levels of glucans [44,50,58]. All these characteristics mean that the use of 100% oat malt for brewing leads to low extract content (due to low DP), low alcohol content and higher viscosity and turbidity, causing filtration problems due to high glucan levels [62]. On the other hand, the use of unmalted oats in different proportions (up to 40%) results in higher wort viscosities as the amount of adjunct increases [57,62]. Despite these drawbacks, the addition of exogenous enzymes when adding proportions of unmalted oats higher than 20% or the use of different types of adjunct, such as oat flour, or the selection of specific crops considerably improves the drawbacks of their use [80,86]. In conclusion, the strategy for the use of oat malt in brewing depends on the used enzyme blends, and in the case of unmalted oats, the selection of specific crops or their pre-processing is important.

Bearing in mind the nutritional and sensory characteristics, beers brewed from 100% oat malt exhibit berry-like flavours and aromas and poor foam stability compared to barley malt beers [50]. Improvements in the sensory quality of beer are also observed with the use of unmalted oats. According to a study by Schnitzenbaumer et al. [80], beers containing 30% and 40% oats were rated higher in terms of aroma and flavour purity than 100% barley malt beers. This is because the oat beers contained considerably lower concentrations of 2-furfural and γ-nonalactone (ageing components), as well as acetaldehyde and a higher content of esters (ethyl acetate and isoamyl acetate) [80]. The same effect has been observed in beers brewed with 100% oat malt [50]. In the case of the combination of oat malt and unmalted oats, beers with a more delicate flavour than 100% oat malt beers are produced as a result of lower levels of esters, higher alcohols and ethanol and the presence of a more diverse amino acid profile [62].

#### 5.2.7. Rye (*Secale cereale* L.)

The application of rye grain in brewing is mainly due to the dry, pungent and astringent characteristics of the grain [51,87] and because it is a source of bioactive compounds (phenolic acids, lignans and alkylresorcinols) [88]. Rye malt produced by standard methods is characterised by a very high viscosity because it contains large amounts of water-extractable arabinoxylans [16,51]. However, this drawback can be overcome by using extended germination times that also increase the activity of amylolytic and proteolytic enzymes [51]. Compared to barley, rye malts have higher levels of extract and starch degrading enzymes (mainly α-amylases) than barley malt [51], so it is often blended with other malted cereals to increase the percentage of fermentable extract [16].

Taking into account nutritional and sensory characteristics, rye malt generally results in sensory-pleasant and dark-coloured beers, largely due to its higher turbidity [22]. Not much information has been collected about the sensory characteristics provided by rye malt. However, as published by Wang in 2017 [51], the different varieties of rye malt on the market (Pale malt, Roasted malt and Crystal malt) provide a spicy and astringent character to the final beer. As for unmalted rye grain, it has been observed that in beers brewed with 100% unmalted rye, colour and brightness decrease, as well as containing higher alcohols and esters levels compared to malted barley beer [72].

#### 5.2.8. Millet (*Eleusine coracana* L. Gaertn., *Pennisetum glaucum* L. and *Eragrostis tef* (Zuccagni))

Millet is employed as a replacement for sorghum and barley malt to brew African craft beer, commonly referred to as opaque beer. The most commonly used millet varieties are finger millet (*Eleusine coracana* L. Gaertn.) and pearl millet (*Pennisetum glaucum* L.) [48,52,89]. The principal technical challenges for the use of millet malt in beer production consist of its small size (which hinders water absorption in steeping), low diastatic power (DP) and low extract yield [79,89]. These factors make it necessary to optimise the malting operation, and the sensory quality of the finished beer product is very different. It is often combined with other malted grains to compensate for this disadvantage.

From a nutritional and sensorial properties, the use of millet malt provides darker beers with sensory properties different from those of standard beers [90]. Millet beer exhibits better foam stability than beers produced with sorghum and barley malts, probably due to its high tannin content [52,91]. One of the most interesting millet subspecies for brewing is Teff (*Eragrostis tef* (Zuccagni)), which in addition to having excellent nutritional properties (antioxidant potential and high mineral concentrations) and usability qualities [92], gives beer unique sensory characteristics. Malted Teff has a malty character with notes of biscuit, vanilla and cereal, while unmalted Teff has a fruitier character [93]. Teff is used like a raw ingredient of gluten-free beer, functional drinks and other gluten-free foods [92]. Of all millet subspecies, pearl millet (*Pennisetum glaucum* L.) is considered the species that gives rise to better sensory characters, as it has less impact on the sensory attributes of beer (appearance, flavour, body and aroma) [94]. In addition, it improves digestibility, sensory and nutritional quality and has a pronounced effect on reducing anti-nutrients [53,54].

### 5.3. Pseudocereals

#### 5.3.1. Buckwheat (Fagopyrum Esculentum Moench)

Buckwheat is potentially suitable for use as a raw ingredient in beer brewing, especially for application in the production of gluten-free beers [4,55]. However, there are some technological issues to be taken into account when brewing buckwheat malt beers. Buckwheat malt has a reduced amylolytic activity and extract yield, reduced filtration rates related to high wort viscosity and fermentation challenges [4,55]. Therefore, it is necessary to use supplementary enzymes to balance the low enzyme activity and enable complete saccharification. If the malting procedure is optimised, buckwheat as the main raw ingredient can be utilised. However, it is always recommended to employ buckwheat in association with other malts or by adding enzymes [35].

Considering the nutritional and sensory characteristics, beers brewed from 100% buckwheat malt develop a particular nutty flavour [4], are darker in colour and have a lower alcohol content than barley malt beers [55,56]. The aroma compounds present in buckwheat beer are in the ranges of a barley malt beer, with the exception of methanol, 2-methylbutanol and 3-methylbutanol. These characteristics make the purity of flavour stand out as a sensory attribute, while bitterness and foam quality are the lowest rated attributes [55]. Using lower proportions of buckwheat malt (20–40%) results in a taste comparable to beer brewed with 100% malted barley, although the bitter intensity is slightly higher, probably due to polyphenols and proteins from buckwheat malt. Beer quality parameters are not altered, resulting in sensory acceptable beers in terms of odour, aroma and taste [4].

Buckwheat is the only pseudocereal that contains rutin, a flavonoid that possesses antioxidant, anti-inflammatory and anticarcinogenic effects, among others [95]. More specifically, it is attributed with higher antioxidant activity than ascorbic acid or chalconaringenin [4]. The use of buckwheat malt in the brewing of lagers has been found to provide beers enriched in rutin. These beers show relatively high antioxidant capacity and oxidative stability during forced ageing, compared to 100% barley malt beer [4]. The increase in total polyphenol composition, and consequently, the antioxidant activity of beer is favoured by milder conditions during malting [4,96].

#### 5.3.2. Quinoa (Chenopodium Quinoa Willd)

Quinoa is presented as a novel adjunct, and its interest in brewing lies in the fact that it is considered a functional food due to its high content of total phenols, high antioxidant activity and high content of minerals and vitamins [97]. The attributes of quinoa malt for use in brewing are: low enzyme activity, low malt extract yield and long saccharification time [55,57]. When unmalted quinoa is used, there are decreases in the extract yield due to the low enzyme content, increased saccharification time and increased viscosity due to the higher glucan content [57,85].

Quinoa in brewing requires the adaptation of brewing procedures, which are still to be developed, and the addition of exogenous enzymes.

In reference to nutritional and sensory properties, malted quinoa beer has a very different appearance from conventional beers due to its almost black colour, nutty aroma, greyish foam and astringent taste. In the volatile compound profile, the presence of pyrazines stands out, which improve the odour perception by providing toasted and nutty aromas. In contrast, taste perception is negatively affected by the high content of metal cations and amino acids [55]. With regard to unmalted quinoa, its use in proportions of up to 40% does not contribute unpleasant characteristics to the beer and even has a positive effect on its overall sensory quality. According to a study by Kordialik-Bogacka et al. published in 2018, all sensory attributes (aroma, taste, mouthfeel, bitterness and carbonation) scored better than beer brewed with 100% barley malt [57].

In terms of the influence of quinoa on the nutritional properties of the beer, it was found that the use of unmalted quinoa serves to enrich the wort with essential metal ions and provides the beer with a higher concentration of these ions. In particular, brewing worts with 30% flaked quinoa added have been found to contain 78% more Mg^2+^ and 20% more Ca^2+^ [57].

#### 5.3.3. Amaranth (*Amaranthus cruenteus*, *Amaranthus hypochondriacus* and *Amaranthus caudatus*)

Amaranth is an adjunct with a high content of essential amino acids, unsaturated fatty acids and minerals. It is significantly richer in Mg^2+^ and Ca^2+^ than barley, which could significantly improve the brew’s yeast yield and fermentation rate [57]. To date, there are few studies on brewing with amaranth. It exhibits a high gelatinisation temperature and saccharification is incomplete [35]. Future studies should be conducted to improve the malting conditions of amaranth, as lower extract yields have been found compared to other gluten-free adjuncts [33]. In any case, its use as an unmalted adjunct may be an innovation in the beer industry that will attract consumer interest [35].

Considering nutritional and sensory properties, the addition of unmalted amaranth, albeit in a very small quantity, seems to provide a great enrichment of brewers’ wort with essential metal ions. According to a research published by Kordialik-Bogacka et al. in 2018 [57], the use of amaranth as an adjunct increased the Mg^2+^/Ca^2+^ ratio (required for the effective transformation of wort sugars into ethanol), as well as the Zn^2+^ and Mg^2+^ content, even when just 10% of the barley malt is substituted.

### 5.4. Other Starchy Products

#### Sweet Potato (*Ipomoea batatas*)

Sweet potato is presented as an adjunct with great potential for use in brewing because it has a high carbohydrate concentration (80–90% of its dry matter) [14,40] and is also a source of β-amylases, which are more heat stable than β-amylases from barley [98]. A study by Etim et al. in 1992 revealed that sweet potato flour has a high diastatic power and can be used to replace sorghum malt in brewing [97]. Another interesting aspect to note is that it has a high content of β-carotene and anthocyanins [14,40] and this may have nutritional implications for beer.

In terms of nutritional and sensory properties, the profile of volatile compounds detected in beer brewed with sweet potato is very similar to most beers. However, depending on the concentration of adjunct and mashing time, the beer has better sensory acceptance and different sensory characteristics [14]. In addition to providing different organoleptic characteristics to beer, the addition of sweet potato to beer shows a positive effect on antioxidant activity, which may be related to its β-carotene content and its synergy with phenolic compounds. Beers containing this adjunct have a higher phenolic content (218–230 mg GAE/L) than pale lagers (110–179 mg GAE/L), but lower than dark beers (230–260 mg GAE/L). It was also shown to significantly increase the β-carotene content (0.2–0.838 mg/100 mL) compared to beer brewed with 100% barley malt (0.003 mg/100 mL) [14]. The use of other sweet potato varieties as an adjunct, such as purple sweet potato, gives the beer a pink colour, and has a good sensory acceptance mainly in colour and flavour. Beer with 30% adjunct has been found to be the most acceptable, with large differences in colour and flavour scores [40]. In addition, this potato variety contains a large number of anthocyanins (mainly peonidin and cyanidin), so increasing the adjunct concentration leads to an increase in the concentration of anthocyanins in the beer. These facts are related to the increased antioxidant capacity of beer, which varies between 6.31–17.06% DPPH, with the antioxidant rates in beer being of the same order of magnitude as those found in fruit juices, teas and wines [40].

## 6. Future Trends

The use of adjuncts is a current trend in the brewing world due to the increasing demand in recent years for more sensory complex beers (in which more intense flavours are perceived) and distinctive beers of higher quality [2]. In particular, the search for “new characteristics” in beer has forced brewers to introduce new adjuncts into the brewing process, both in industrial and craft brewing.

Focusing on the starchy adjuncts discussed in this article (cereals, pseudocereals and tubers), a trend that has been developing for some time now is the brewing of non-gluten beers. Among the non-gluten starchy adjuncts from which non-gluten beers can be produced are cereals, such as sorghum, maize, rice and millet, and pseudocereals, such as buckwheat, amaranth and quinoa [75,84]. Sorghum is the most widely researched and prominent cereal for the production of non-gluten beers [99].

More recently, research has investigated how the incorporation of extruded adjuncts influences beer brewing. In terms of organoleptic characteristics, a higher number and concentration of aroma compounds have been detected in beers using extruded grains as adjuncts than in those brewed with unextruded adjuncts [15,100,101]. A study published in 2018 shows a higher content of aroma compounds in extruded corn starch beer than in cooked corn starch beer [101]. In another study by Zhang et al. in 2019, using extruded black rice, novel compounds (nerolidol, geraniol and geranylgeraniol) were detected that were not present in unextruded black rice beers. Therefore, the use of extruded grains can be interesting from a sensory point of view for brewing [15].

The use of adjuncts that enrich beer with antioxidant compounds is also an important trend at present. Among them, buckwheat is presented as a source of flavonoids (mainly rutin), and its use considerably increases the antioxidant activity of beer [4].

A tuber such as sweet potato, used as an adjunct, increases the flavonoid and β-carotene content [14]. Recently, Vieira et al. [14] have shown that Beauregard sweet potato is a potentially valuable adjunct in the beer production process, as it exhibited successful values for physicochemical variables, such as pH, viscosity, bitterness and colour, higher alcohol content, higher levels of phenols and flavonoids, and had a considerable acceptance in the sensory analysis. In addition, the Beauregard sweet potato resulted in a higher β-carotene content in the final beer and may have influenced the antioxidant activity of the final product. These results can also be used as a basis for future research on enhancing the functionality and sensory features of Beauregard sweet potato beer.

Yeast (*Saccharomyces cerevisiae*), fungus (*Aspergillus oryzae*) and bacteria (*Lactobacillus plantarum*) can be used to produce cassava beer with probiotic properties [102].

We suggest to carry out research on the use of the yeast *Saccharomyces cerevisiae* in association with two lactic acid bacteria, *Lactobacillus plantarum* and *Weissella cibaria* to improve the quality of beers produced using adjuncts. Similar studies have been carried out on rice wine production in South Korea [102].

*Torulaspora delbrueckii*, *Saccharomyces cerevisiae* and *Metschnikowia pulcherrima* could be used to produce different sorghum beers with specific characteristics such as fruity and phenolic character, reduced volatile acid production and improvement of aroma, flavour and colour characteristics upon fermentation [103]. Infrared spectroscopy can be used to analyse and characterise the sorghum flour chemical composition. A single ATR-FTIR scan is now capable of delivering information on the main constituents of sorghum flour, including starch, proteins, lipids and phenolic acids [89]. Digital image processing could be an interesting solution to conventional laboratory methods for the analysis of crude protein, tannin and total phenolic constituents in sorghum grain [104].

Zdaniewicz et al. [105] examined the potential of tritordeum to be used as an adjunct in the brewing industry. The alcohol yield of the beer brewed with tritirdeum, the actual extract, and the metal ion content were at a similar range to the control sample. Additionally, the utilisation of tritordeum in a large proportion range did not lead to any technological challenges in the latter phases of the brewing process.

In an investigation of the use of adjuvants in brewing, it is necessary to mention the developments in commercial enzymes and their application. Due to the close relationship between the use of adjuncts in the brewing industry and the use of commercial enzymes, it is likely that advances in biotechnology to improve known enzyme functions, as well as to design new enzymes with different functionalities, will be associated with an increase in the use of adjuncts in beer production [106].

It will also enable the production of premium adjuncts with added benefits tailored to the needs of brewers and may even lower the manufacturing cost of adjuncts, thereby reducing the cost of beer production. In fact, the monitored application of heat is the usual method of inducing a similar phenomenon and is routinely exploited in the manufacture and processing of brewing adjuncts. Several studies have shown that high-pressure leads to gelatinisation of cereal starches and boosts the efficiency of starch hydrolysis by α- and β-amylases [107]. Therefore, it would be worth investigating whether high-pressure processing, which is an expensive technology, might be used in the research on the development of addition agents for enhanced processability of brewers’ adjuncts in the beer industries.

## 7. Conclusions

The vast majority of beers on the market today contain a wide variety of starch adjuncts, some of which can reduce production costs or boost several beer quality attributes, such as colour or flavour, although not all starch adjuncts have the right attributes to be used in brewing.

The use of adjuncts may lead to beneficial beer organoleptic characteristics: some of these changes can be seen in colour (dark or pink beers) or in aromas and flavours (fruity aromas and more intense flavours). Wheat or rye malts are commonly used, while other adjuncts such as maize or rice are more commonly used in the form of starches or grits. The use of extruded adjuncts has recently acquired a particular interest.

On the nutritional side, the use of non-conventional starch adjuncts, such as black rice, buckwheat or sweet potato, leads to an increase in the polyphenol content of the beer, and thus, in its antioxidant capacity. Sorghum and sweet potato can be considered two potential raw materials for the production of specific beers (gluten-free beer, antioxidant-rich beer, etc.).

However, very often, the utilisation of novel raw materials leads to difficulties in beer production, such as the necessity to use external enzymes, excessive saccharification or filtering time, very low wort extract and thereby low alcohol content in beer, etc. Therefore, the possibility of novel raw ingredients as adjuncts for beer processing could be a very interesting line of research for the development of beers with different organoleptic characteristics.

## Figures and Tables

**Table 1:** Common brewing adjuncts available.

Adjunct Form	Cereals
Whole cereal	Barley, buckwheat, maize, sorghum, triticale, wheat
Grits	Barley, maize, rice, sorghum
Flaked	Barley, corn, oats, rice
Torrified/micronised	Barley, corn, wheat
Extrusion cooked	Maize, rice, sorghum, wheat
Flour/starch	Cassava, corn, potato, rice, soya, sorghum, wheat
Syrup	Barley, corn, potato, sucrose, wheat
Malted cereals	Oats, rye, sorghum, wheat
Malted pseudo-cereals	Buckwheat, quinoa

**Table 2:** Range of total starch, amylose (AM), amylopectin (AP) and protein content in the five predominant cereal brewing grains [26].

Cereal	Range in Starch Content (%)	Range in AM Content (%)	Range in AP Content (%)	Range in Protein Content (%)
Barley	50–60	22–27	78–73	8–20
Corn	75–80	24–31	76–69	6–10
Rice	75–87	23–30	77–70	6–10
Sorghum	65–75	22–27	78–73	6–15
Wheat	65–70	22–27	78–73	9–20

**Table 3:** Characteristics of starchy adjuncts used in breweries.

Starchy Adjunct	Gelatinisation Temperature (°C)	Adjunct Extract (%)	Malt Extract (%)	Diastatic Power (WK Unit) *
Wheat	52–66 [16]	75 [16]	85.7 [16]	405 [22]
Barley	58–66 [35]	70 [16]	76–88 [35]	200–416 [35]
Maize	62–80 [35]	78 [16]	68–68.75 [35]	77 [35]
Rice	67–91 [35]	84 [16]	64.3–77.8 [35]	19–62 [35]
Sorghum	69–80 [35]	82 [16]	68 [35]	72–101 [35]
Oats	52.6–62 [35]	72 [16]	62.1 [35]	82–124 [35]
Rye	50–62 [16]	74 [16]	89.2 [22]	177 [35]
Millet	54–80 [35]	sd **	59.6–68.9 [35]	40–61 [35]
Buckwheat	65.4–72 [35]	sd **	61.9–65.3 [35]	72 [35]
Quinoa	64 [35]	sd **	37.7 [35]	61 [35]
Amaranth	64–74 [35]	sd **	88.6–91.1 [35]	sd **

*: WK = Windisch Kolbach **: sd = standard deviation.

**Table 4:** Starch adjuncts used in brewing, correlation with beer type and effects on sensory/nutritional properties.

Starch Adjunct	Type of Adjunct	Type of Beer	Effect on Sensory/ Nutritional Properties	Reference
Wheat	Chocolate Malt Crystal Malt Dark Malt	Ale/Lager	More pronounced malt aromas Darker colour Fuller bodied	[41]
	Wheat Flour	Ale	Pale in colour Lower foam stability Higher intensity of grain odour	[42]
Barley	Grinded barley	Lager	Pale colour More intense bitter taste More astringency	[32,43,44]
Maize	Maize malt	Ale	Slightly bitter taste Less foam stability	[7]
	Blue maize malt	Lager	Higher anthocyanin content	[45]
	Grinded maize	Lager	Pale colour Decrease in polyphenol content Decrease in antioxidant activity	[9,11]
Rice	Pale rice malt	Ale/Lager	Light vanilla flavour Thick foam Flat sensory profile	[46]
	Caramel rice malt Dark rice malt	Ale	Darkening of the colour (amber colour) Higher intensity of malt, caramel and vanilla aromas Higher antioxidant activity	[47]
	Extruded black rice flour	Lager	Higher content of essential amino acids	[15]
Sorghum	Sorghum malt	Ale/Lager	Slightly tart flavour Fruity aromas Darker colour	[16,41,48,49]
Oats	Oat malt	Lager	Fruity aromas Berry flavour Less foam stability	[50]
	Grinded oatmeal	Lager	Improved aroma and flavour purity Reduced foam stability Darker colour	[13]
Rye	Crystal Malt Pale Malt Toasted Malt	Ale	Spicy and sour character Stronger flavours Darkens the beer	[41,51]
Millet	Millet malt (pearl millet, finger millet)	Lager	Darkens the colour yeasty aroma Raw grain flavour	[49,52,53,54]
	Teff malt	Ale	Malty character Biscuit, vanilla and cereal notes	[55]
	Grinded teff	Ale	Fruity character Banana, apple and tropical aromas	[55]
Buckwheat	Buckwheat malt	Lager	Darker colour Nutty flavour Higher antioxidant activity	[4,55,56]
Quinoa	Quinoa Malt	Lager	Almost black in colour Greyish foam Toasted aromas Nutty flavour	[55]
	Quinoa flakes	Lager	Darker colour Higher concentration of essential minerals (Mg^2+^ and Ca^2+^)	[57]
Amaranth	Grinded amaranth	-	Increase in the ratio of Mg^2+^ and Ca^2+^. Increase in Zn^2+^ and Mg^2+^ content	[58]
Sweet potato	Beauregard sweet potato flakes	Ale	Increased β-carotene content Increased antioxidant activity	[14]
	Purple sweet potato flakes	Ale	Pink colour Increased anthocyanin content Increased antioxidant capacity	[22]
